# Retroperitoneal teratoma with somatic malignant transformation: A papillary renal cell carcinoma in a testicular germ cell tumour metastasis following platinum-based chemotherapy

**DOI:** 10.1186/1471-2490-13-9

**Published:** 2013-02-12

**Authors:** Nina Zeh, Peter J Wild, Peter K Bode, Glen Kristiansen, Holger Moch, Tullio Sulser, Thomas Hermanns

**Affiliations:** 1 Department of Urology, University Hospital Zürich, Zürich, Switzerland; 2 Institute of Surgical Pathology, University Hospital Zürich, Zürich, Switzerland; 3 Institute of Pathology, University of Bonn, Bonn, Germany

**Keywords:** Retroperitoneal teratoma, Malignant transformation, Germ cell tumour metastasis, Renal cell cancer

## Abstract

**Background:**

Malignant transformation describes the phenomenon in which a somatic component of a germ cell teratoma undergoes malignant differentiation. A variety of different types of sarcoma and carcinoma, all non-germ cell, have been described as a result of malignant transformation.

**Case presentation:**

A 33-year-old man presented with a left testicular mass and elevated tumour markers. Staging investigations revealed retroperitoneal lymphadenopathy with obstruction of the left ureter and distant metastases. Histopathology from the left radical orchiectomy showed a mixed germ cell tumour (Stage III, poor prognosis). The ureter was stented and four cycles of cisplatin, etoposide and bleomycin chemotherapy administered. After initial remission, the patient recurred four years later with a large retroperitoneal mass involving the renal vessels and the left ureter. Left retroperitoneal lymph node dissection with *en-bloc* resection of the left kidney was performed.

Histopathology revealed a germ cell tumour metastasis consisting mainly of mature teratoma. Additionally, within the teratoma a papillary renal cell carcinoma was found. The diagnosis was supported by immunohistochemistry showing positivity for AMACR, CD10 and focal expression of RCC and CK7. There was no radiological or histo-pathological evidence of a primary renal cell cancer.

**Conclusions:**

To the best of our knowledge, malignant transformation into a papillary renal cell carcinoma has not been reported in a testicular germ cell tumour metastasis following platinum-based chemotherapy. This histological diagnosis might have implications for potential future therapies. In the case of disease recurrence, renal cell cancer as origin of the recurrent tumour has to be excluded because renal cell carcinoma metastases would not respond well to the classical germ cell tumour chemotherapy regimens.

## Background

Teratoma is a tumour of embryonic origin and belongs to the group of non-seminomatous germ cell tumours (GCT) [1]. Primarily, it occurs most often in the testis or ovary but may also appear in other body sites such as the central nervous system, mediastinum or retroperitoneum [2]. Non-seminomatous GCT can be pure teratoma but mixed forms with other non-seminomatous histologies occur more frequently. However, teratoma is often the only component that remains histologically detectable in residual lesions after platinum-based chemotherapy for metastatic mixed non-seminomatous GCT.

Teratoma typically contains different types of tissue derived from all three germinal layers, the endoderm, mesoderm and ectoderm. It can be of cystic or solid appearance and depending on the degree of differentiation seen it is classified as mature or immature teratoma [1]. The immature form is characterised by somatic differentiated cells in an early ‘embryonal’ stage. A mature teratoma consists of fully differentiated somatic tissues such as cartilage, epidermis, nerve tissue, glands or even complex organs. Mature renal tissue has rarely been reported to be a part of mature teratoma [3].

Somatic malignant transformation (MT) refers to malignant de-differentiation of teratoma components [2]. It is a well-described phenomenon, characterized by differentiation of pluripotent teratoma cells into somatic tumour cells [2,4-7]. Usually MT occurs in adult patients with metastatic GCT disease and can arise either in the primary or metastatic site of the tumour [6]. Sarcoma is the most frequently described malignant histology but a variety of other tumour entities including enteric adenocarcinoma, primitive neuroectodermal tumour (PNET) and leukaemia have also been found [2,4-6]. To the best of our knowledge, MT into papillary renal cell cancer has never been reported in a testicular GCT metastasis following platinum-based chemotherapy.

## Case presentation

A 33-year-old man presented with a left-sided, painless scrotal mass suspicious for testicular cancer. Scrotal ultrasound showed a 3 cm mass of heterogeneous echogenicity in the left testicle with irregular, poorly defined borders. Serum tumour markers were elevated with an alpha-fetoprotein (AFP) of 28135 μg/l, beta-human chorionic gonadotropin (ß-HCG) of 19700 U/l and lactate dehydrogenase (LDH) of 4421 U/l. Staging thoraco-abdominal computed tomography (CT) showed bulky retroperitoneal lymphadenopathy, left hydronephrosis, metastases to both liver and lung, as well as mediastinal and supraclavicular lymphadenopathy.

The patient underwent left inguinal orchiectomy and routine biopsy of the contra-lateral testis in a secondary referral centre. A double-J stent was inserted into the left ureter.

Histo-pathological examination of the left testis revealed a mixed GCT consisting of two-thirds classical seminoma and one-third non-seminomatous tumour with embryonal carcinoma and yolk sac tumour components. In the adjacent parenchyma intra-tubular germ cell neoplasia was found. The right testis biopsy showed normal testicular tissue without intra-tubular germ cell neoplasia. The tumour stage was determined to be pT1 cN3 cM1b S3 (TNM; [7]) and clinical stage IIIC poor prognosis group (Lugano classification; [8]).

**Figure 1:**
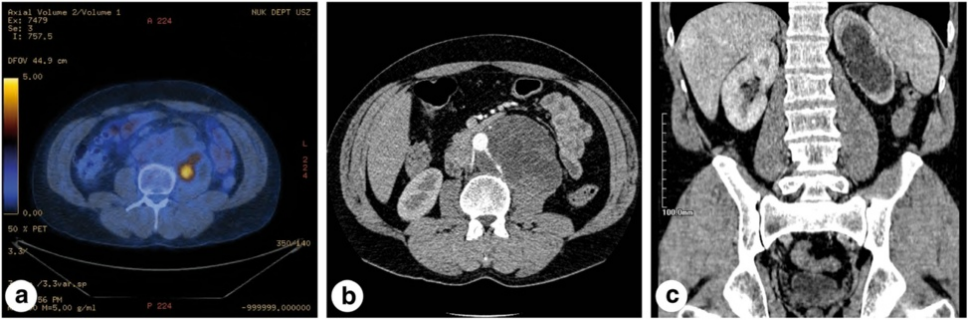
**Pre-operative positron emission tomography showing a large retroperitoneal mass with a centrally located metabolic activity of 2.4 cm in diameter (a).** Computed tomography (CT) with the large retroperitoneal mass and involvement of the renal vessels (**b**). A kidney tumour was detectable neither in the right nor in the left hydronephrotic kidney (**c**).

Subsequently, the patient was discharged to a community oncologist and treated with four cycles of cisplatin, etoposide and bleomycin chemotherapy. Five months after completing chemotherapy all serum tumour markers normalised. Regular follow-up thoraco-abdominal CTs showed residual retroperitoneal and supra-clavicular lymphadenopathy, along with sub-centimetre pulmonary and liver masses. As these post-chemotherapy lesions were small and did not show interval growth, surgical resection was not performed. Four and a half years after orchiectomy the patient was referred to our academic tertiary referral centre with a newly enlarging retroperitoneal mass. Fluorodeoxyglucose positron emission tomography (FDG-PET) had been performed at the community and this demonstrated a partially FDG-avid retroperitoneal mass infiltrating the left renal vessels, left ureter and abdominal aorta (Figure 1a-c). Tumour markers remained normal. The hepatic, pulmonary, mediastinal and supra-clavicular lesions were unchanged. Both kidneys appeared normal on abdominal CT and FDG-PET.

Left retroperitoneal lymphadenectomy with *en-bloc* resection of the left kidney and the infrarenal aorta was performed. An aorto-bi-iliacal graft was inserted.

### Histopathological findings

**Figure 2:**
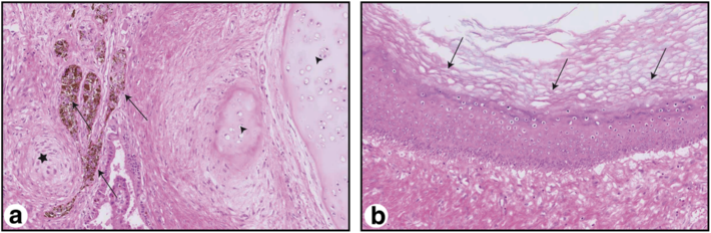
**Hematoxylin and eosin (HE) staining of the mature teratoma containing pigmented epithelium (arrows a), cartilage (arrowhead a), peripheral nerves (asterisk a) as well as squamous epithelium (arrows b).** Magnification: 100x.

Histology of the *en-bloc* resected specimen revealed a retroperitoneal GCT metastasis with a maximum diameter of 12 cm. The metastasis consisted of mature cystic teratoma containing peripheral nerves, cartilage and respiratory, squamous and pigmented epithelium (Figure 2a and b).

**Figure 3:**
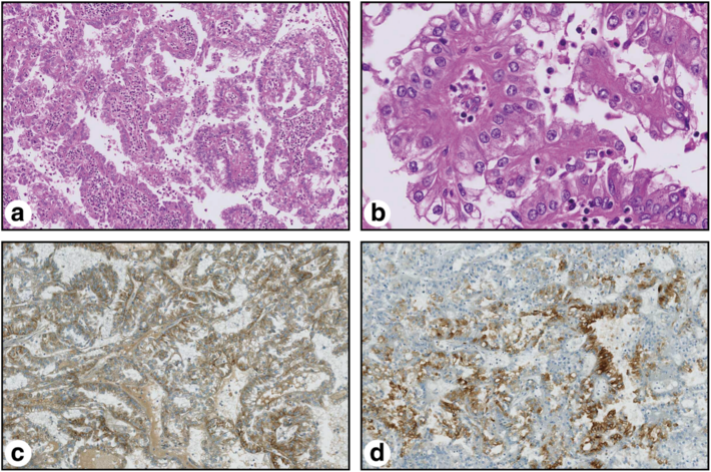
**Overview (a) and detailed view (b) of the papillary renal cell carcinoma within the teratoma.** The HE staining shows the typical papillary histoarchitecture with pleomorphic nuclei, fine-granular chromatin and eosinophilic cytoplasm (**a**, **b**). Immunohistochemistry (**c**, **d**) with positive immunoreactivity for alpha-methylacyl-CoA racemase (AMACR) (**c**) and focal positivity for renal cell carcinoma antigen (RCC) (**d**). Magnification: 100x (**a**,**c**,**d**), 400x (**b**).

Within the teratoma, a 2.5 cm lesion with the typical appearance of a papillary renal cell carcinoma was found. Areas of papillary and tubular histoarchitecture were seen (Figure 3a). The cell nuclei appeared to be pleomorphic containing fine-granular chromatin. The cytoplasm of these cells was eosinophilic (Figure 3b).

Immunohistochemistry showed strong positivity for alpha-methylacyl-CoA racemase (AMACR, Figure 3c), CD 10 (not shown), focal expression of renal cell carcinoma antigen (RCC; Figure 3d) and cytokeratin 7 (CK 7; not shown). Vimentin and AFP were negative (not shown). Histologically, no renal cell cancer was found in the removed left kidney.

### Follow-up

No additional chemotherapy was administered after surgery. Currently, 2 years after retroperitoneal lymphadenectomy, the patient remains progression-free with stable disease showing no further tumour growth, metabolic PET-activity or tumour marker elevation.

## Discussion

Somatic MT develops in 3-6% of chemotherapy-*naïve* patients with metastatic GCT containing teratomous components [4,9]. After platinum-based chemotherapy the incidence of MT increases up to 14% [4]. Histologically, various types of sarcoma and carcinoma have been identified in GCT with MT. The most commonly MTs reported are rhabdomyosarcoma, PNET and adenocarcinoma. Leiomyosarcoma, liposarcoma, chondrosarcoma, Non-Hodgkin’s lymphoma and leukaemia are found less frequently [2,3,6]. Renal tumour differentiation is rare. Nephroblastoma (Wilms tumour) have been described [6] and there is a single report of MT into papillary renal cell carcinoma in a primary retroperitoneal and chemotherapy-*naïve* GCT [10]. To the best of our knowledge, MT into papillary renal cell cancer within a testicular GCT metastasis following platinum-based chemotherapy has not been previously documented.

It is highly unlikely that the papillary renal cell cancer found was a metastasis originating from the kidneys. We believe the papillary renal cell carcinoma resulted from MT of the teratoma for several reasons. Firstly, the lesion was located within the teratoma. Secondly, both kidneys had normal appearance on multiple abdominal CTs and FDG-PET. Finally, there was no histological evidence of renal cell cancer in the left kidney that was removed *en bloc*. There was also no family history of papillary renal cell cancer. In cases with more clinical ambiguity, fluorescence in situ hybridisation (FISH) analysis for 12p amplification can be used to confirm the germ-cell origin of somatic-type tumours of uncertain histogenesis [11].

The characteristic morphology and the immunohistochemical profile confirmed the diagnosis of papillary renal cell cancer. Papillary renal cell cancer is differentiated from other subtypes of renal cell cancer by using CK7 and AMACR immunohistochemical markers [12]. Most papillary renal cell carcinomas are positive for CK7, whereas clear cell renal cell cancer shows either negative or only a focal or expression pattern [13]. A panel of immunohistochemical markers, including CD10, RCC, and vimentin, has been proposed for the identification of renal origin of a metastatic tumour [12,14]. Identification of the most common chromosomal aberrations in papillary renal cell cancer (trisomy of chromosome 7 and 17 as well as loss of Y chromosome [15]) were not necessary in our case due to convincing histological and immunohistochemical features.

The role of FDG-PET in evaluating residual masses after chemotherapy differs between seminomatous and non-seminomatous GCTs [16]. FDG-PET can discriminate between viable tumour and fibrosis or necrosis in post-chemotherapy residuals of seminomatous GCT [17,18]. Therefore, FDG-PET is recommended to decide whether surveillance or surgical therapy is the treatment of choice in patients with residuals > 3 cm [19]. However, in the setting of a non-seminoma post-chemotherapy mass FDG-PET is not recommended [19]. Although it can detect viable tumour in residual masses, it cannot discriminate mature teratoma from fibrosis or necrosis [20]. Therefore FDG-PET is not helpful to decide if surgical therapy is necessary or not in these patients.

In our case of non-seminomatous GCT, FDG-PET was performed contradictory to standard guidelines prior referral to our centre. Histo-pathological examination revealed that the FDG-avid lesion within the retroperitoneal teratoma represented the papillary renal cell cancer. It has been reported that FDG-PET may have a role in detecting metastasis of renal cancer. However this is usually in the setting of clear cell rather than papillary carcinoma [21].

Due to the wide spectrum and unpredictability of tumour types that occur in MT, the utility of FDG-PET in the setting of MT remains unknown and requires further investigation. Tissue diagnosis is still the gold standard in determining presence of viable malignancy and discriminating between GCT and other types of tumours.

The occurrence of MT is known to worsen the prognosis of GCT, particularly in metastatic disease [6,22-24]. One reason for this is that many of the tumours found after MT are highly aggressive (i.e. sarcoma, PNET) [25,26]. If MT remains undetected, a conventional platinum-based chemotherapy approach may fail in treating the new tumour type developed. This may additionally delay diagnosis of MT and lead to worse outcome [27,28].

Complete surgical resection, if technically possible, should remain the gold standard of therapy as it will achieve the most reliable histopathologic diagnosis, and has been shown to improve the prognosis of patients with MT in metastatic GCT [2,3,6,27]. If MT is found, histological evaluation is mandatory for all synchronously or metachronously detected metastases. In advanced cases with documented spread of the transformed histologic subtype, systemic therapies targeted to the identified tumour type should be considered [6,9,27,29].

## Conclusions

To the best of our knowledge, MT into papillary renal cell carcinoma has not been reported in the setting of metastatic testicular germ cell tumour following platinum-based chemotherapy. Our case highlights the importance of complete surgical resection and histological diagnosis of residual masses after chemotherapy of metastatic non-seminomatous GCTs to allow tailoring of potential subsequent therapies to the transformed tumour type.

### Consent

Written informed consent was obtained from the patient for publication of this Case report and any accompanying images. A copy of the written consent is available for review by the Series Editor of this journal

## Abbreviations

GCT: Germ cell tumour; MT: Malignant transformation; PNET: Primitive neuroectordermal tumour; AFP: Alpha-fetoprotein; ß-HCG: Beta-human chorionic gonadotropin; LDH: Lactate dehydrogenase; CT: Computed tomography; FDG-PET: Fluorodeoxyglucose positron emission tomography; AMACR: Alpha-methylacyl-CoA racemase; RCC: Renal cell carcinoma antigen; CK7: Cytokeratin 7.

## Competing interests

The authors declare that they have no competing interests.

## Authors’ contributions

NZ was responsible for acquisition of data, drafting of the manuscript and preparation of the figures. PJW and PKB were responsible for the histopathologic investigations and contributed to the pathological interpretation of data. GK and HM were responsible for the concept and interpretation of the pathological investigations and revised the manuscript for important intellectual content. TS operated the patient and also revised the manuscript for important intellectual content. TH was responsible for the concept, design, acquisition and interpretation of data and revision of the manuscript. All authors read and approved the final manuscript.

## Pre-publication history

The pre-publication history for this paper can be accessed here:

http://www.biomedcentral.com/1471-2490/13/9/prepub
